# Cardiac imaging for the prediction of sudden cardiac arrest in patients with heart failure

**DOI:** 10.1016/j.heliyon.2023.e17710

**Published:** 2023-06-28

**Authors:** Francesca De Lio, Alessandro Andreis, Giulia De Lio, Matteo Bellettini, Stefano Pidello, Claudia Raineri, Guglielmo Gallone, Gianluca Alunni, Simone Frea, Massimo Imazio, Davide Castagno, Gaetano Maria De Ferrari

**Affiliations:** a Division of Cardiology, Città della Salute e della Scienza di Torino University Hospital, University of Torino, Turin, Italy; b Cardiology Unit, Cardiothoracic Department, University Hospital “Santa Maria della Misericordia”, Udine, Italy

**Keywords:** Cardiac imaging, Sudden death, Heart failure, ICD, Speckle tracking, Arrhythmias, Diastole, Cardiac magnetic resonance, Ventricular fibrillation, Ventricular tachycardia

## Abstract

The identification of heart failure (HF) patients at risk for arrhythmic sudden cardiac arrest (SCA) is a major challenge in the cardiovascular field. In addition to optimal medical treatment for HF, implantable cardioverter defibrillator (ICD) is currently recommended to prevent SCA in patients with reduced left ventricular ejection fraction (LVEF). The indication for an ICD implantation, in addition to HF etiology, New York Health Association (NYHA) class and life expectancy, mainly depends on LVEF value at echocardiography. However, the actual role of LVEF in the prediction of SCA has recently been debated, while newer multimodality imaging techniques with increased prognostic accuracy have been developed. Speckle tracking imaging allows the quantification of mechanical dispersion, a marker of electrophysiological heterogeneity predisposing to malignant arrhythmias, while advanced cardiac magnetic resonance techniques such as myocardial T1-mapping and extracellular volume fraction assessment allow the evaluation of interstitial diffuse fibrosis. Nuclear imaging is helpful for the appraisal of sympathetic nervous system dysfunction, while newer computed tomography techniques assessing myocardial delayed enhancement allow the identification of focal myocardial scar.

This review will focus on the most modern advances in the field of cardiovascular imaging along with its applications for the prediction of SCA in patients with HF. Modern artificial intelligence applications in cardiovascular imaging will also be discussed.

## 1 Introduction

Arrhythmic sudden cardiac arrest (SCA) due to ventricular fibrillation or sustained ventricular tachycardia accounts for approximately half cardiovascular deaths, representing a major public health issue [1]. SCA occurs more frequently in patients with heart failure (HF), especially those with more extensive myocardial fibrosis and adverse remodeling [2]. In addition to guideline-directed medical treatments for HF, implantable cardioverter defibrillator (ICD) is recommended to prevent SCA in patients with HF and a reduced ejection fraction [[3], [4], [5]]. However, many ICD recipients do not receive any appropriate ICD therapy for decades, while exposed to potential complications or a non-negligible risk of inappropriate ICD therapy [[6], [7], [8]]. Furthermore, despite the well-known benefit of ICD among HF patients with ischemic heart disease (IHD), its role among patients with non-ischemic heart disease (NIHD) is still debated [7]. In patients with HF, left ventricular ejection fraction (LVEF) assessment is crucial since a cut-off of 35% or lower is currently required before considering ICD for primary prevention of SCA. However, the value of LVEF has been widely questioned as a single reliable predictor of ventricular arrhythmias (VA) causing SCA, while in the last few years newer imaging techniques have been developed with increased prognostic significance [9,10].

This review will discuss the role of multimodality imaging techniques for the prediction of SCA in patients with HF.

## 2 Echocardiography

Myocardial injury may result in tissue fibrosis, represented by a focal scar, which is often the case after an acute myocardial infarction, or diffuse interstitial, as commonly observed in NIHD. Myocardial fibrosis is a major substrate for ventricular arrhythmias (VA), as it causes electrical dispersion, laying ground for the initiation and maintenance of re-entrant VA [11].

### 2.1 Ejection fraction

In 1980s, lacking technologies able to measure scar size and characteristics, systolic function estimated with 2D-echocardiography (i.e., LVEF) emerged as an indirect marker of scar burden. The most recent European Society of Cardiology guidelines provide different recommendations for ICD implantation for primary prevention of SCA in patients with ≥1 year of life expectancy according to the HF etiology. In IHD, they provide a Class I recommendation in patients with symptomatic heart failure (NYHA class II or III) and a LVEF≤35% despite ≥3 months of optimal medical therapy (OMT) and a Class II recommendation in asymptomatic patients (NYHA class I) and LVEF≤30% despite ≥3 months of OMT [12]. A class II recommendation is also provided in patients with IHD, LVEF≤40% despite ≥3 months of OMT, non-sustained VTs and inducible sustained VTs at electrophysiological study [12]. For NIHD, they provide a class II recommendation in patients with NYHA class II or III and a LVEF≤35% despite ≥3 months OMT [12]. The LVEF cut-off of 35% was chosen based on the inclusion criteria of the major randomized trials demonstrating the efficacy of ICD in the reduction of all-cause mortality and cardiac death [[13], [14], [15]]. However, this criterion has been later questioned from several authors, not only because of the poor reproducibility of echocardiography-assessed LVEF but also for its limited sensitivity and specificity [16,17]. For instance, in 2013 Narayanan et al. assessed a cohort of 2093 patients with SCA, showing that 68% of them had an LVEF>35% and would have been considered ineligible for ICD implantation [7,18].

### 2.2 Global longitudinal strain

**Fig. 1:**
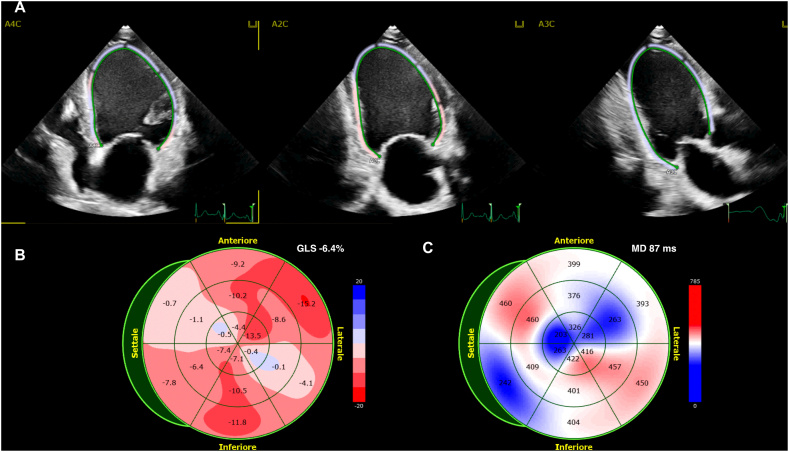
A B C – 1A. Echocardiography based speckle tracking analysis (GLS and MD) in a patient with HF, admitted for sustained VA. 1B. Bull's eye view of segmental longitudinal strain of left ventricle. 1C. Bull's eye view of segmental time to peak systolic strain of left ventricle.

Speckle tracking echocardiography is a modern technique which can accurately quantify the extent and timing of systolic deformation [19,20], as shown in Fig. 1A and B. GLS not only is more accurate and reproducible than LVEF in quantifying LV systolic function, but its impairment indirectly reflects myocardial fibrosis [21]. GLS proved to be an independent predictor of VA in both patients with previous myocardial infarction and patients with NIHD and its value was confirmed also in patients with LVEF >35% [10]. Haugaa et al. found in a prospective study on 94 patients with NIHD and LVEF <50% that GLS was a significant predictor of arrhythmic events, with greater accuracy as compared with LVEF (respectively with an AUC of 0.82, 95% CI 0.70–0.95 vs. 0.72, 95% CI 0.57–0.87) [22]. A study by Kalra et al. on patients with HF reported that worsening of systolic function assessed with GLS was associated with an increased risk of SCA, specifically a 58% increase in the risk for every 1-percentage point GLS decrease (hazard ratio 1.58, 95%CI: 1.12 to 2.22). A more recent application of speckle tracking echocardiography includes layer specific GLS assessment. One of the possible application fields of this technology is the setting of arrhythmogenic cardiomyopathy (ACM). Indeed, arrhythmic risk stratification in ACM remains a matter of debate and LVEF is a poor predictor, with a remarkable incidence of SCA in patients with preserved or mildly impaired LVEF, particularly in certain genotypes. In a recent study of 45 subjects with borderline diagnosis of arrhythmogenic cardiomyopathy, epicardial GLS was superior to traditional, endocardial GLS in the identification of patients with arrhythmias, reflecting the typical earlier subepicardial involvement [23].

### 2.3 Mechanical dispersion

Beyond GLS, speckle tracking echocardiography allows the quantification of mechanical heterogeneity of left ventricular contraction. Mechanical dispersion (MD) is calculated as the standard deviation of the time to peak longitudinal strain in the 16-segments of the left ventricle [24], as showed in Fig. 1C. MD represents the extent of electrical dispersion (ED) due to strands of myocardial scar. Electrical dispersion is a key point in the physiopathology of reentry, and it manifests itself on surface electrocardiogram as dispersion of QRS complexes (dQRS), QTc (dQTc) and Tpeak-Tend (dTpe) intervals. All these elements, representing depolarization or repolarization heterogeneity, proved to be associated with an increased risk of VTs [78]. MD, being an epiphenomenon of ED, also proved to be an independent predictor of arrhythmic events in both NIHD [10,21,25] and IHD [26] cohorts. Recently, a large retrospective observational multicenter study was performed to assess the long-term prognostic value of MD in patients with moderate or severe LVEF impairment [27]. This study enrolled 939 consecutive patients with a LVEF≤45%. MD ≥ 75 ms was a significant predictor of VA events, defined as symptomatic VA or ICD appropriate intervention or death (hazard ratio 9.45, 95% CI 4.75–18.81), while both LVEF, age and etiology were not. Interestingly, among patients with MD < 75 ms, there was no difference in VA events between patients with severely reduced LVEF ≤35% and those with moderately impaired LVEF (36% to 45%), further supporting the potential of MD for risk-stratification.

Fig. 1 shows assessment of global longitudinal strain (GLS) in a patient with IHD with a GLS of −6.4%. The panel A displays regional strain map superimposed on the two-dimensional echocardiographic images in apical four-chamber (A4C), apical two-chamber (A2C), and apical three-chamber (A3C) views. The panel B shows regional longitudinal strain (bull's eye view) for each segment of a 16-segment model of the left ventricle. Panel C displays the time (ms) between aortic valve opening and peak longitudinal strain for each segment and the global MD.

### 2.4 Diastolic dysfunction and left atrial strain

The assessment of diastolic dysfunction as a predictor of SCA emerged only recently, despite a strong rationale and previous findings from experimental studies [28,29]. In a recent cohort study, Pezawas et al. showed that, among 210 patients (120 with IHD, 60 with NIHD and 30 with normal LVEF), those with grade-III diastolic disfunction had the highest risk of arrhythmic death or resuscitated cardiac arrest (hazard ratio 3.52, 95% CI 2.00–6.22). This was found for both patients with LVEF≤35% (p < 0.001) and patients with LVEF >35% (p = 0.014) [30].

Left atrial strain is a modern technique to assess diastolic dysfunction with increased accuracy across different clinical situations [31]. In a recent study by Carluccio et al. [32], including 405 patients with LVEF ≤40%, an impaired LA reservoir function defined as a reduced peak atrial longitudinal strain, not only was associated with worse left ventricular systolic and diastolic function, but also with increased risk of the all-cause death or HF hospitalization (hazard ratio 1.38 per 1-unit decrease, 95% CI 1.05–1.84). In another study of 357 patients with HF with preserved ejection fraction, peak atrial longitudinal strain was a predictor of the composite endpoint of SCA, cardiac death or HF hospitalization (hazard ratio 0.96 per 1-unit increase, 95% CI 1.05–1.84) [33].

Table 1 summarizes some of the major studies that used echocardiography in the prediction of sudden cardiac death in HF patients.

**Table 1:** Echocardiography in the prediction of sudden cardiac death in HF patients.

Author, Year	n	Study Design	Inclusion criteria	Echocardiographic parameter	Endpoint and main results	Follow-up
Moss A.J., 2002	1232	Prospective, randomized controlled trial	Prior Myocardial infarction and LVEF ≤30%	LVEF ≤30%	Death from any cause (19.8% vs. 14.2%, p < 0.001)	20 months
Bardy G. H., 2005	2521	Prospective, randomized controlled trial	Heart failure (ischemic or non-ischemic), LVEF ≤35%, NYHA II-III	LVEF ≤35%	Death from any cause (29% vs 22%, p < 0.001)	45,5 months
Kadish A., 2004	458	Prospective, randomized controlled trial	NIHD, symptomatic heart failure, LVEF <36% and non-sustained ventricular tachycardia or at least 10 premature ventricular complexes per hour	LVEF <36%	Death from any cause (14.1% vs. 7.9%, p < 0.001)	29 months
Haugaa K.H., 2013	569	Prospective, multicenter	Prior Myocardial infarction (>40 days)	Global longitudinal strain < - 16%	Arrhythmic events (sustained ventricular tachycardia, ventricular fibrillation, and SCA). C-statistics AUC 0.71	30 months
Haugaa K.H., 2013	569	Prospective, multicenter	Prior Myocardial infarction (>40 days)	Mechanical dispersion >75 ms	Arrhythmic events (sustained ventricular tachycardia, ventricular fibrillation, and SCA). (20% vs 1% p < 0.001). C-statistics AUC 0.75	30 months
Haugaa K.H., 2012	94	Prospective, observational	NIHD, dilated cardiomyopathy and LVEF <50%	Global longitudinal strain < −7,1%	Death from any cause and arrhythmic events (sudden cardiac arrest, sustained ventricular tachycardia, appropriate therapy from ICDs)	22 months
Haugaa K.H., 2012	94	Prospective, observational	Non ischemic dilated cardiomyopathy and LVEF <50%	Mechanical dispersion >72 msec	Death from any cause and arrhythmic events (sudden cardiac arrest, documented sustained ventricular tachycardia, appropriate therapy from ICDs implanted for primary prophylaxis, and syncope with probable cardiac cause) (44% vs 6% p < 0.001)	22 months
Perry R., 2020	939	Retrospective, observational, multicenter	Heart failure with LVEF ≤45% (at least 40 days post- hospital admission for an MI or HF event and on optimal medical therapy)	LVEF ≤35%	Ventricular arrhythmias, defined as first incidence of arrhythmic death (SCD), symptomatic VA (either sustained VT and/or VF), or appropriate ICD therapy (55% vs 41% p < 0.001)	24 months
Perry R., 2020	939	Retrospective, observational, multicenter	Heart failure with LVEF ≤45% (at least 40 days post- hospital admission for an MI or HF event and on optimal medical therapy)	Global longitudinal strain ≥ - 14%	Ventricular arrhythmias, defined as first incidence of arrhythmic death (SCD), symptomatic VA (either sustained VT and/or VF), or appropriate ICD therapy (90% vs 81% p < 0.001)	24 months
Perry R., 2020	939	Retrospective, observational, multicenter	Heart failure with LVEF ≤45% (at least 40 days post- hospital admission for an MI or HF event and on optimal medical therapy)	Mechanical dispersion ≥75 ms	Ventricular arrhythmias, defined as first incidence of arrhythmic death (SCD), symptomatic VA (either sustained VT and/or VF), or appropriate ICD therapy (91% vs 47% p < 0.001)	24 months
Pezawas T., 2020	210	Prospective, observational	Heart failure (IHD, NIHD) and patients with normal LVEF	Diastolic dysfunction grade III	Arrhythmic death or resuscitated cardiac arrest (58% vs 37% p < 0.01 vs 21,5% vs 4,5% p < 0.001)	10 years
Carluccio E., 2018	405	Prospective, observational	LVEF ≤40%, stable sinus rhythm, no/moderate-to-severe aortic stenosis or degenerative mitral regurgitation, no planned revascularization procedures, and optimized medical therapy since 3 months.	Left atrial reservoir function: peak atrial longitudinal strain ≤12,9%	All cause death and hospitalization for HF (29,8% vs 15,9% vs 4,7% p < 0.001)	30 months

## 3 Cardiac Magnetic Resonance

### 3.1 Late gadolinium enhancement

**Fig. 2:**
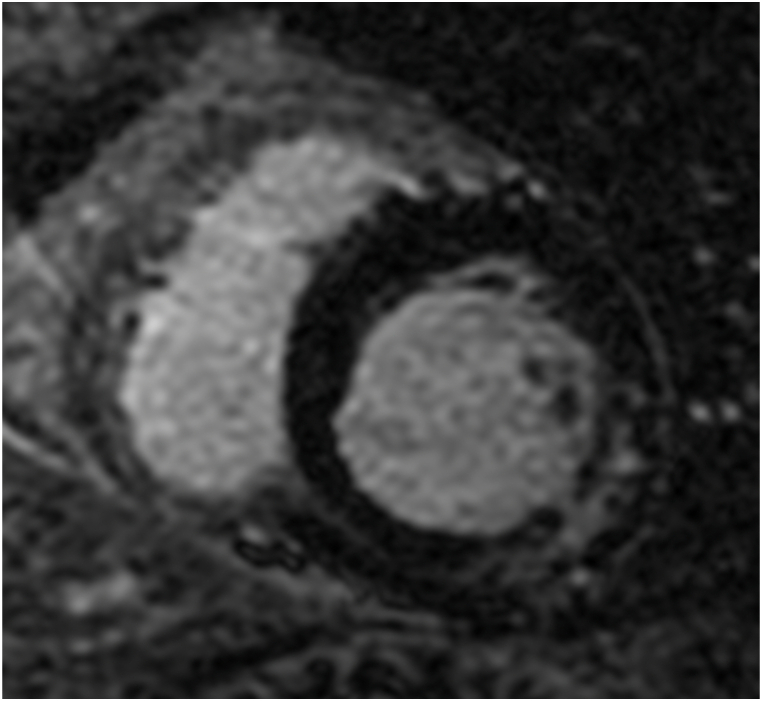
Cardiac Magnetic Resonance of a patient with ventricular arrhythmias. Left ventricular short axis view showing intramyocardial late gadolinium enhancement.

Late gadolinium enhancement (LGE) assessed with cardiac magnetic resonance (CMR) is an accurate technique for the characterization of myocardial tissue and the detection areas of focal myocardial fibrosis, appearing hyperintense on delayed CMR imaging [34,35]. Fig. 2 shows an example of a midwall striae of fibrosis. Most patients with a prior myocardial infarction show myocardial LGE on CMR imaging [36]. In fact, in the setting of IHD, both LGE presence and extent have been associated with death or appropriate ICD therapy for sustained VAs [37,38]. In addition, LGE-CMR allows the characterization of the ischemic scar, constituted by a central infarct core zone and a peri-infarct gray zone. The gray zone is the region where the viable myocardium is intertwined with tissue fibrosis, creating an ideal substrate for VA [39,40]. In a study of 91 patients with IHD referred for ICD implantation, the extent of the gray-zone was the only significant predictor of appropriate ICD therapies (hazard ratio 1.49/10 g, 95% CI 1.01–2.20), while total infarct size, LVEF and LV volumes were not [41]. A significant association between the occurrence of VA and the extent of gray zone was also reported in another study of 162 patients with ST-segment elevation myocardial infarction treated with primary percutaneous coronary intervention, during a 1-year follow-up [42]. However other studies reported contradictory results and therefore the role of gray zone is still debated [43]. These contrasting data might be due to lack of consensus on the methodology of scar and gray zone quantification and the use of different study methodologies [44]. LGE was reported as a strong independent predictor of VAs, SCA and ICD appropriate therapy, even when adjusted for other clinical or functional parameters, in prospective and retrospective studies [[45], [46], [47], [48], [49]]and meta-analyses [50,51].

At variance with IHD, in the setting of NIHD LGE is not a constant feature, being identified in 45–77% of affected patients according to different studies [37,52]. However, a meta-analysis by Becker et al. [51] including 34 studies on a total of 4554 patients with NIHD, showed that LGE-positive patients had an increased risk of VAs, SCA and ICD appropriate interventions (hazard ratio 4.52, 95% CI 3.41–5.99). In the NICM-SCAR study [44] published in 2021, including 1020 patients with NIHD and a LVEF <50%, only myocardial scar assessed with LGE-CMR provided strong independent and incremental prognostic value for risk stratification for SCA and arrhythmic events (p = 0.001) as opposed to LVEF ≤35% (p = 0.57). Furthermore, in NIHD, LGE-CMR can be helpful in the differential diagnosis of the underlying myocardial disease, according to the type of enhancement pattern. The most common pattern in NIHD is linear midwall, subepicardial or patchy without coronary artery territory distribution [53]. Midwall fibrosis has been well recognized as a negative prognostic marker in NIHD patients, implying a higher risk of SCA [54,55]. In the recent DERIVATE registry, midwall fibrosis in >3 segments was the strongest predictor of arrhythmic events in NIHD patients [56]. LGE-CMR imaging could then represent a promising technique to identify the subgroup of NIHD patients who might benefit more from ICD implantation.

Moreover, recent studies further explored the characterization of scar by postprocessing LGE sequences and its correlation with arrhythmic risk. In 2021, Acosta et al. analyzed the correlation between scar characterization and an arrhythmic endpoint in 217 patients (39.6% with IHD) with a class I indication for CRT. Among these patients, in addition to the presence and the extension of scar, the heterogeneity of scar (expressed as border zone mass/scar mass ratio) and the border zone channel mass were the strongest predictors of the primary composite endpoint of appropriate ICD therapy or SCD, representing the ideal substrate for reentry arrhythmias [57].

An important and still unclear question is whether there is a quantitative relationship between scar extent and cardiac events and whether does exist an optimal threshold of scar extent able to discriminate high-risk patients, thus needing adequate therapeutic interventions. Some studies reported that scar extent is associated with arrhythmic events in both IHD and NIHD [55,58,59]. In a recent meta-analysis of 19 studies, Disertori et al. reported a significant association between LGE extent and arrhythmic endpoints. Among 2850 patients included in the meta-analysis, 423 experienced arrhythmic events. This occurred in 23.9% of patients with LGE positive CMR versus 4.9% of patients with negative LGE imaging. Ten of the studies included in the meta-analysis also reported a statistically significant increase of the arrhythmic risk with increasing LGE extension, with no significant differences between IHD and NIHD [60].

In the last few years there has been a growing interest in a peculiar distribution pattern of LGE: the so-called ring-like left ventricular scar, defined by the presence of at least three contiguous sub-segments with LGE at the subepicardial or mid-wall layer in the same slice. This pattern has been reported to be a common feature of left dominant arrhythmogenic cardiomyopathy [61], partially overlapping with the arrhythmogenic subtypes of dilatative cardiomyopathy (DCM). A recently published retrospective study [52] of 157 patients with NIHD investigated the relationship between ring-like LGE and VA. Among all patients, 77% showed LGE, among which 21% showed a ring-like LGE pattern. After a median of 13 ± 7 months of follow-up, arrhythmic events defined as a composite of sustained VT, VF, SCA and appropriate ICD intervention were more common in patients with ring-like (hazard ratio 11.75, 95% CI 2.66–51.92) and multifocal LGE (hazard ratio 5.55, 95% CI 1.21–25.44). A limitation of LGE imaging is that it allows the detection of focal fibrosis but not interstitial diffuse fibrosis.

Fig. 2 shows a left ventricle short-axis CMR image of a patient with NIHD with a midwall striae of LGE at the inferolateral wall.

### 3.2 Parametric mapping

Further advances in CMR imaging techniques allowed the detection of diffuse myocardial fibrosis, by means of myocardial T1-mapping and extracellular volume fraction assessment. Indeed, diffuse myocardial fibrosis results in longer T1 native relaxation times compared with normal myocardium [62], and in shortened T1 relaxation times after contrast-media injection [63]. In a prospective longitudinal study of 130 IHD and NIHD patients [64], myocardial native T1 time was an independent predictor of the primary composite endpoint of VA or ICD appropriate intervention (hazard ratio 1.10, 95% CI 1.04 -1-16). In another prospective study, Claridge et al. [65] performed CMR-based T1-mapping in patients undergoing ICD implantation. In this study on 130 patients, the T1 native value was the only independent predictor of appropriate ICD therapy in the NIHD cohort (hazard ratio 1.09, 95% CI 1.04–1.14). In IHD patients, on the contrary, the strongest predictor of arrhythmic events was the presence of a gray-zone. In NIHD, ECV was shown to predict cardiovascular death, hospitalization for HF and sustained symptomatic VA [66].

## 4 Computed tomography

Myocardial delayed enhancement (MDE) with computed tomographic (CT) imaging has been demonstrated to accurately display scar tissue as compared with pathological specimens and LGE-CMR [67,68]. However, as compared to CMR, because of the lower accuracy and increased radiation exposure, MDE-CT imaging has not been widely adopted in clinical routine.

## 5 Nuclear imaging

Nuclear perfusion imaging using Single Photon Emission Computed Tomography (SPECT) with technetium 99 m or positron emission tomography (PET) with rubidium-82, 15O-labeled water or 13N-ammonia are useful techniques to assess the presence of reversible or non-reversible (i.e., scar) myocardial perfusion defects. Reversible myocardial perfusion defects at nuclear imaging identify areas of viable tissue with inducible myocardial ischemia. Inducible myocardial ischemia and viable dysfunctional myocardium may create a vulnerable substrate predisposing to VA. In relation to inducible myocardial ischemia, in a retrospective analysis of SPECT imaging in a large cohort of patients with IHD, summed stress score was significantly associated with increased risk of SCA [69]. In a recent retrospective analysis of 170 patients with IHD [70], myocardial flow reserve assessed by PET imaging was the only significant predictor of a composite primary endpoint including SCA.

The ability of nuclear imaging in the visualization and quantification of underlying pathophysiological processes predisposing to VTs could be particularly useful in NIHD, in which the benefit of implantation of ICD in primary prevention is still debated [71]. The study of coronary flow reserve has revealed that coronary vascular dysfunction is highly prevalent among patients with NIHD, thus providing another element to help risk stratification in patients with HF. A recent study [72] included 510 patients with IHD or NIHF with LVEF ≤45% referred for rest/stress myocardial perfusion PET imaging. Abnormal coronary flow reserve (CFR) was observed in most patients of both populations; moreover, patients with CFR ≤1.65 in both IHD and NIHD experienced higher primary endpoint (a composite of cardiac death, aborted SCD, late revascularization or HF hospitalization).

In addition to myocardial ischemia, hibernating myocardium was shown to be vulnerable to VA [73]. The PAREPET (prediction of arrhythmic events with positron emission tomography) study showed no significant association between the amount of hibernating myocardium and SCA, however this result could be affected by a very low prevalence of hibernating myocardium, due to the broad use of revascularization in the contemporary era [74].

The pivotal role of sympathetic imbalance for the development of VA is well-known, especially in the setting of myocardial ischemia [75]. Indeed the inhomogeneity of myocardial innervation assessed both with SPECT imaging using 123 meta-iodo-benzyl guanidine (123I-mIBG) and with PET imaging using 11C-*meta*-hydroxyephedrine (11C-HED) was shown to be a strong predictor of SCA [[76], [77], [78]]. In the prospective ADMIRE-HF (AdreView Myocardial Imaging for Risk Evaluation in Heart Failure) study [78], in a population of 961 patients with IHD and NIHD with LVEF ≤35%, a reduced HMR (heart-to-mediastinum ratio) of 123I-mIBG was the strongest predictor of the primary composite endpoint of cardiac death, arrhythmic events and hospitalization for chronic HF in both populations. Arrhythmic events were significantly more common in patients with HMR <1.6. Data were also confirmed in the PARAPET study [74] showing that each 1% of the volume of denervated myocardium results in an 6% increase of SCA.

## 6 Conclusions

Recent advances in cardiac imaging techniques led to the identification of a great number of imaging parameters associated with increased risk of SCA in patients with HF. It appears time to overcome the limitations posed by echocardiography-based LVEF assessment, and to implement a multimodal-imaging approach, which is key to best predict patients at increased risk of SCA.

## Author contribution statement

All authors listed have significantly contributed to the development and the writing of this article.

## Data availability statement

The authors do not have permission to share data.

## Special issue

This item belongs to the item group IG000035.

## Declaration of competing interest

The authors declare that they have no known competing financial interests or personal relationships that could have appeared to influence the work reported in this paper.
